# Different types of soluble fermentable dietary fibre decrease food intake, body weight gain and adiposity in young adult male rats

**DOI:** 10.1186/1743-7075-11-36

**Published:** 2014-08-14

**Authors:** Clare L Adam, Patricia A Williams, Matthew J Dalby, Karen Garden, Lynn M Thomson, Anthony J Richardson, Silvia W Gratz, Alexander W Ross

**Affiliations:** 1 Rowett Institute of Nutrition and Health, University of Aberdeen, Greenburn Road, Bucksburn, Aberdeen AB21 9SB, UK

**Keywords:** Dietary fibre, Beta-glucan, Fructo-oligosaccharide, Pectin, Cellulose, Fermentation, Satiety, Adiposity, Body weight regulation

## Abstract

**Background:**

Dietary fibre-induced satiety offers a physiological approach to body weight regulation, yet there is lack of scientific evidence. This experiment quantified food intake, body weight and body composition responses to three different soluble fermentable dietary fibres in an animal model and explored underlying mechanisms of satiety signalling and hindgut fermentation.

**Methods:**

Young adult male rats were fed *ad libitum* purified control diet (CONT) containing 5% w/w cellulose (insoluble fibre), or diet containing 10% w/w cellulose (CELL), fructo-oligosaccharide (FOS), oat beta-glucan (GLUC) or apple pectin (PECT) (4 weeks; n = 10/group). Food intake, body weight, and body composition (MRI) were recorded, final blood samples analysed for gut satiety hormones, hindgut contents for fermentation products (including short-chain fatty acids, SCFA) and intestinal tissues for SCFA receptor gene expression.

**Results:**

GLUC, FOS and PECT groups had, respectively, 10% (*P* < 0.05), 17% (*P* < 0.001) and 19% (*P* < 0.001) lower food intake and 37% (*P* < 0.01), 37% (*P* < 0.01) and 45% (*P* < 0.001) lower body weight gain than CONT during the four-week experiment. At the end they had 26% (*P* < 0.05), 35% (*P* < 0.01) and 42% (*P* < 0.001) less total body fat, respectively, while plasma total glucagon-like peptide-1 (GLP-1) was 2.2-, 3.2- and 2.6-fold higher (*P* < 0.001) and peptide tyrosine tyrosine (PYY) was 2.3-, 3.1- and 3.0-fold higher (*P* < 0.001). There were no differences in these parameters between CONT and CELL. Compared with CONT and CELL, caecal concentrations of fermentation products increased 1.4- to 2.2-fold in GLUC, FOS and PECT (*P* < 0.05) and colonic concentrations increased 1.9- to 2.5-fold in GLUC and FOS (*P* < 0.05), with no consistent changes in SCFA receptor gene expression detected.

**Conclusions:**

This provides animal model evidence that sustained intake of three different soluble dietary fibres decreases food intake, weight gain and adiposity, increases circulating satiety hormones GLP-1 and PYY, and increases hindgut fermentation. The presence of soluble fermentable fibre appears to be more important than its source. The results suggest that dietary fibre-induced satiety is worthy of further investigation towards natural body weight regulation in humans.

## Background

There is growing interest in targeting increased satiety as a potential countermeasure to the current global obesity epidemic, often embracing pharmacological and surgical approaches (eg [1-3]). However, an alternative physiological approach is to use food constituents that naturally increase satiety and reduce overall caloric intake. Dietary fibre is one such food constituent variously associated with decreasing appetite and body weight, but data from human trials remain equivocal [4-8]. This may be resolved in the first instance using laboratory animals where there is complete control over the diet and the opportunity to collect gut tissue samples in which to explore underlying molecular mechanisms. Studies in rats and mice have indeed shown that diets containing increased amounts of dietary fibre result in lower body weight and/or adiposity; however, effects on food intake and satiety are inconsistent, in part due to the variability in study designs, for example in terms of fibre type, duration of feeding, dose rate, and age and phenotype of the experimental animals [9-13]. Herein the aim was to control for the variations found in previous studies by quantifying responses to different types of soluble dietary fibre within the same study, using a fixed duration of feeding, a fixed dose rate and conventional young adult male rats. While results from this animal model may not apply directly to solve the obesity epidemic in humans, they may contribute to the scientific knowledge base leading towards that ultimate goal.

Dietary fibre is a general term describing indigestible carbohydrates that can be broken down by bacterial fermentation in the large intestine and includes insoluble molecules such cellulose that are poorly fermented but also soluble molecules that tend to be more highly fermented [10,14,15]. This study utilised three commonly-occurring yet contrasting types of soluble fermentable dietary fibre, with cellulose included as an insoluble control fibre for comparison. Beta-glucan is a highly polymerised branch-structured glucose polysaccharide found in cereals and bran, pectin is a highly polymerised galacturonic acid polysaccharide present in fruit and vegetables, and fructo-oligosaccharide (FOS) is a fructose polymer with a contrastingly low degree of polymerisation, present in fruit and vegetables as well as in artificial sweeteners. These soluble fibres also demonstrate different physicochemical attributes, for example pectin and beta-glucan have high viscosity whereas FOS has negligible viscosity, while all are highly fermentable [10,14,15]. Choice of these fibres was influenced by existing evidence for responses to their inclusion in diets for *ad libitum*-fed rats and mice in a wide range of study designs. Thus dietary inclusion of oat beta-glucan at 7% for 6 weeks decreased caloric intake and there were trends towards decreased body weight gain and decreased fat pad weights in adult diet-induced obese mice [9] but given at 5% for 6 weeks had no effect on food intake or body weight while decreasing epididymal fat pad weights in growing rats [10]. Food intake and growth rate were decreased in weanling rats given dietary pectin at 8% for 14 days [11] whereas food intake increased and body weight was unaffected in young rats given 5% pectin for 21 days in another unrelated study [12]; effects on body composition were not reported in these studies. In growing rats, dietary inclusion of FOS at 10% for 3-4 weeks decreased food energy intake, body weight gain and adipose tissue mass [13] but at 5% for 6 weeks in another study only decreased epididymal fat pad mass without affecting intake or body weight [10], while 6% of a similar carbohydrate type, galacto-oligosaccharide, in the diet for 3 weeks also reduced epididymal fat-pad weight [16]. Guided by these previous studies, the present study used normal outbred young adult rats, a standard dietary fibre inclusion rate of 10% and a duration of 4 weeks to compare and quantify intake, body weight and body composition responses to different soluble fibres for the first time within the same study.

Several decades of research have identified the key role played by gastrointestinal peptide hormones in the control of food intake and hunger/satiety [17]. Thus ghrelin secreted from the stomach stimulates food intake while cholecystokinin (CCK), peptide tyrosine tyrosine (PYY) and glucagon-like peptide-1 (GLP-1) from the intestine signal satiety and inhibit food intake [18]. It is postulated that the satiating effects of dietary fibre are mediated at least in part by these gut hormones since, for example, FOS feeding is associated with increased circulating GLP-1 and decreased ghrelin in rats [19] and oat beta glucan increases PYY secretion in diet-induced obese mice [9,20], but there are no reports on the effects of dietary pectin on these gut hormones. Gut hormone secretion was therefore assessed in our model from both plasma concentrations (ghrelin, CCK, PYY and GLP-1) and gut tissue gene expression (PYY and GLP-1). Since these hormones are secreted by enteroendocrine cells within the gut mucosa, it was also pertinent to investigate how these cells sense the presence of dietary fibre in the gut lumen. The products of dietary fibre fermentation are organic acids, mainly the short-chain fatty acids (SCFAs) acetate, propionate and butyrate, but also others such as succinate and lactate [21]. The presence of SCFA-activated receptors in colonic mucosa provides grounds for implicating SCFA sensing in the stimulation of gut satiety hormones [22]. Furthermore SCFA activation of free fatty acid receptors (ffar)2 and ffar3 has been associated with stimulation of PYY and GLP-1 secretion in human and rodent models [23-25]. The succinate receptor (sucnr1) is also expressed in intestinal mucosa [26] but has not been linked to gut hormone secretion. The present study therefore examined in the animal model concentrations of fermentation products (including SCFA) in the lower gut contents and gene expression for SCFA receptors in the lower gut tissue.

This experiment investigated the hypothesis that soluble dietary fibre from different sources decreases voluntary food intake, body weight and body fat mass in normal healthy young adult male rats and that the increased satiety may be brought about by increased amounts of fermentation products in the lower gut signalling via SCFA-activated receptors to stimulate gut satiety hormone secretion. The article reports that these soluble dietary fibres did indeed decrease food intake, weight gain and adiposity, and these changes were associated with increased hindgut fermentation and increased circulating concentrations of satiety hormones GLP-1 and PYY.

## Methods

### Diets

All diets during the study were purified, based on the AIN-93 M diet (American Society for Nutrition, Bethesda, MD, USA) for the maintenance of adult rats, and were made and supplied by Special Diet Services Ltd, Witham, Essex, UK (Table 1): The control diet contained 5% w/w cellulose (CONT), an additional control diet contained 10% w/w cellulose (CELL), and the experimental diets contained 10% w/w fructo-oligosaccharide (FOS), oat beta-glucan (GLUC) or apple pectin (PECT) (Table 1). During the final week before the experiment all rats were habituated to the purified CONT diet.

**Table 1:** **Composition of experimental diets**^**1**^

	**Diet**				
	**CONT**	**CELL**	**FOS**	**GLUC**	**PECT**
Ingredients (% w/w)					
Maize starch	46.57	41.57	41.57	41.57	41.57
Maltodextrin	15.5	15.5	15.5	15.5	15.5
Sucrose	10	10	10	10	10
Casein	14	14	14	14	14
Soyabean oil	4	4	4	4	4
AIN-93 Mineral mix	3.5	3.5	3.5	3.5	3.5
AIN-93 Vitamin mix	1	1	1	1	1
Choline bitartrate	0.25	0.25	0.25	0.25	0.25
L-cystine	0.18	0.18	0.18	0.18	0.18
Cellulose	5	10	0	0	0
FOS^2^	0	0	10	0	0
Beta-glucan^3^	0	0	0	10	0
Pectin^4^	0	0	0	0	10
ME^5^ (MJ/kg)	15.7	15.2	15.2	15.2	15.2
GE^6^ (MJ/kg)	17.2	17.4	17.2	17.1	17.1
Composition (% w/w)					
Dry matter	91.9	92.2	92.2	91.9	92.0
Total carbohydrate^7^	63.5	58.5	59.5	65.3	57.5
Total nitrogen^8^	2.0	2.0	2.0	2.1	2.1
Total fat^9^	4.5	5.0	5.2	4.7	4.7
NSP^10^- soluble	0.50	0.46	8.78^11^	7.74^12^	4.23
NSP^10^- insoluble	2.02	3.45	0.45	0.46	0.62

^1^All diets were based on AIN-93 M diet (American Society for Nutrition, Bethesda, MD USA) and manufactured by Special Diet Services Ltd. (Witham, Essex UK).

^2^Fructo-oligosaccharide (Higher Nature FOS Powder; Revital Ltd., Ruislip, Middlesex UK).

^3^Oat beta-glucan (Cambridge Commodities Ltd., Ely, Cambridgeshire UK).

^4^Apple pectin (Solgar Apple Pectin; Revital Ltd.).

^5^ME, metabolisable energy calculated from Atwater Fuel Energy of diet components.

^6^GE, gross energy determined using Gallenkamp Adiabatic Bomb Calorimeter.

^7^Total carbohydrate by standard acid-hydrolysis followed by colorimetric glucose determination.

^8^Total nitrogen determined using VarioMax CN Analyser.

^9^Total fat measured using standard acid-hydrolysis and gas chromatography.

^10^NSP, non-starch polysaccharides, analysed by the Englyst procedure [27], using gas chromatography and spectrophotometry to measure constituent sugars, and including

^11^fructose detection for FOS diet by ultra performance liquid chromatography.

^12^GLUC diet analysed specifically for beta-glucan by assay kit (K-BGLU; Megazyme, Bray, Co. Wicklow, Ireland).

### Animals, experimental procedure and tissue collection

All animal experimental procedures conformed to the UK Home Office Animal (Scientific Procedures) Act 1986, met institutional and national guidelines for the care and use of animals and were approved by local ethical review at the University of Aberdeen Rowett Institute of Nutrition & Health. The animal rooms were maintained at 21 ± 2°C and 55 ± 10% relative humidity, cages contained sawdust bedding with shredded paper for nesting and plastic tunnels for further enrichment, water was available *ad libitum* and the lighting regime was a standard 12 h light, 12 h dark.

After 1 week acclimatisation to individual housing while on CONT diet, young adult outbred male Sprague Dawley rats (12 weeks old, 467 ± 6.0 g; from Charles River Laboratories UK) were randomly allocated to weight-matched groups and offered the pelleted experimental diets *ad libitum* for 4 weeks (n = 10/group). Voluntary food intake was measured daily (by weighing food into the hopper and weighing uneaten food 24 h later) and body weight was measured twice a week. Body composition was measured in conscious rats at the start (0 week) and end (4 weeks) of the experiment by magnetic resonance imaging (MRI; EchoMRI 2004, Echo Medical Systems, Houston, TX, USA), which measures the masses of fat and lean tissues in live animals using nuclear magnetic resonance (NMR) technology (validated in [28,29]): the fat reading includes all of the fat molecules in the body expressed as equivalent weight of canola oil, and the lean reading is a muscle mass equivalent of all the body parts containing water, excluding fat, bone and substances that do not contribute to the NMR signal such as hair and claws.

After the final MRI scan, rats were euthanised by decapitation under general inhalation anaesthesia (isoflurane; IsoFlo®, Abbott Animal Health, Maidenhead, Berkshire, UK), approximately 1-3 h after lights on. Final (trunk) blood samples were collected into chilled tubes containing EDTA as anti-coagulant and a peptidase inhibitor cocktail containing general protease inhibitor (cØmplete; Roche Diagnostics Ltd, Burgess Hill, West Sussex, UK) and dipeptidyl peptidase-4 inhibitor (Ile-Pro-Ile; Sigma), centrifuged immediately at 3000 g for 10 min, then plasma was stored at -20°C until analysis. The gut was dissected out for sampling the contents from caecum and colon and tissue from distal ileum and proximal colon. Caecum and colon contents were stored at -20°C until analysis. Tissue samples were immersed in RNAlater (QIAGEN, Crawley, UK) for 5 days at 4°C and then stored at -80°C until analysis.

### Plasma analyses

Plasma samples were analysed by commercial radioimmunoassay kits according to the manufacturers’ instructions. Total GLP-1 was measured by kit GLP1T-36HK (Merck Millipore, Billerica, MA, USA; lower detection limit 3 pM) which detects all forms of GLP-1, and active GLP-1 was measured by kit GLP1A-35HK (Merck Millipore; lower detection limit 3 pM) which detects only the biologically active form of GLP-1, i.e. GLP-1(7-36) amide or GLP-1(7-37). PYY was measured by kit RMPYY-68HK (Merck Millipore; lower detection limit 15.6 pg/ml), which detects both of the circulating biologically active forms of PYY, namely PYY(1-36) and PYY(3-36). Total ghrelin was measured by kit RK-031-31 (Phoenix Pharmaceuticals Inc., Burlingame, CA, USA; detection range 10-1280 pg/ml) which detects the combined octanoylated (bioactive) and des-octanoylated forms, and bioactive CCK (26-33) (non-sulfated) was measured by kit RK-069-04 (Phoenix Pharmaceuticals Inc.; detection range 10-1280 pg/ml).

### Analysis of fermentation products

The concentrations of SCFA and other organic acids produced by bacterial fermentation in caecum and colon contents were determined by capillary gas chromatography using the method developed by Richardson *et al.*[30]. Briefly, samples were diluted with distilled water (1/4) and 2-ethylbutyric acid (5 mmol/L) was added as internal standard. Samples were then extracted in diethyl ether, derivatised with N-tert-butyldimethylsilyl-N-methyltrifluoroacetamide and analysed on Agilent GC HP-1 capillary columns. This method detects formate, acetate, propionate, butyrate, iso-butyrate, valerate, iso-valerate, lactate and succinate.

### RNA isolation and real-time qPCR

Total RNA was extracted from the gut tissue samples using RNeasy® Mini Kit (QIAGEN, Crawley, UK). Briefly, weighed samples were homogenised using Zirconia beads (BioSpec, Bartlesville, USA) and a Precellys®24 bead-based homogeniser (Bertin Technologies, Ann Arbor, USA), the homogenates were centrifuged in RNeasy spin columns and on-column DNase digestion was conducted before eluting the RNA. Total RNA was quantified by measuring the absorbance at 260 nm using a NanoDrop spectrophotometer and purity assessed by measuring the ratio of absorbance at 260 and 280 nm. Overall quality was also assessed using an Agilent Bioanalyzer (Agilent Technologies, Santa Clara, USA) and all samples had RIN values >8.9. RNA was then stored at -80°C. The synthesis of cDNA from the RNA was carried out using a high capacity cDNA kit and completed using a GeneAmp® PCR System 9700 thermal cycler (both from Applied Biosystems, Warrington, UK).

Relative gene expression analysis was carried out in line with the Minimum Information for Publication of Quantitative Real-Time PCR Experiments (MIQE) guidelines [31]. Initially, the expression of three candidate reference genes β-actin (beta-actin), B2M (beta-2-microglobulin) and Tbp (TATA box binding protein), together with ffar2, was determined by qPCR using a 7500 Fast Realtime PCR System (Applied Biosystems). B2M had the closest level of expression and similar PCR efficiency to ffar2 and was therefore chosen as the most appropriate reference gene for this study. Commercially available PCR primers were used (QIAGEN, Crawley, UK) for Tbp (reference # QT01605632), β-actin (QT00193473), B2M (QT00176295), ffar2 (QT00384860), ffar3 (QT01299144), sucnr1 (QT00454496), GLP-1 (QT00192661), and PYY (QT02352637). RNA abundance was measured by qPCR with a 7500 Fast Realtime PCR System (Applied Biosystems) using the SYBR green detection method.

### Statistics

Daily food intakes and twice weekly body weight data were analysed by repeated measures ANOVA (General Linear Model with time, diet and their interaction as factors; Minitab version 16, Minitab Inc., State College, PA). Cumulative food intake, initial and final MRI data, changes in body weight, fat mass and lean mass during the experiment and final plasma hormone concentrations were analysed by one-way ANOVA (Minitab). Pair-wise group comparisons were then made by the Tukey method. Pearson’s product–moment correlation was used to examine the relationships between food intake and bodyweight gain, changes in fat and lean mass, and plasma gut satiety hormone concentrations (Minitab). For qPCR data, group-wise comparisons of gene expression ratios were performed using the public domain program Relative Expression Software Tool (REST; http://www.gene-quantification.info/) [32]. The gene expression values were normalised to the reference gene B2M and the data are presented as median expression levels relative to the level in the CONT group. Overall, *P* values of 0.05 or less were considered to be statistically significant.

## Results

### Food intake, body weight and body composition

**Figure 1:**
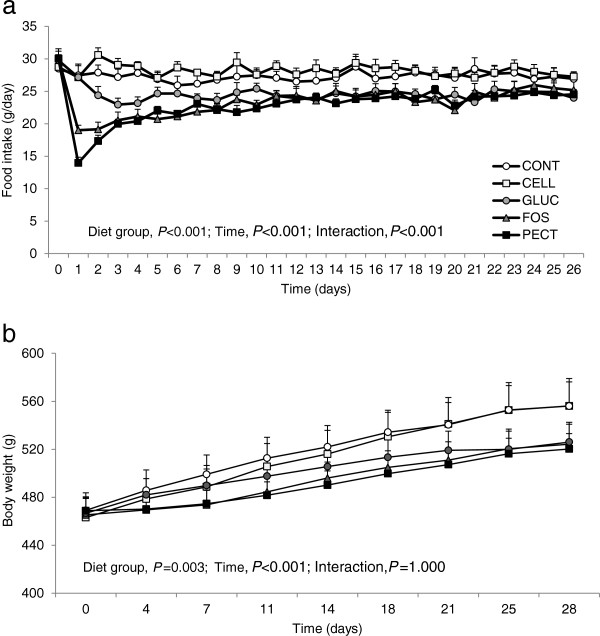
**Food intakes and body weights. (a)** Daily voluntary food intake and **(b)** twice weekly body weights in rats given diets containing different dietary fibres for 4 weeks. Diets contained 5% w/w cellulose (CONT) or 10% w/w fibre as cellulose (CELL), oat beta-glucan (GLUC), fructo-oligosaccharide (FOS) or apple pectin (PECT). Values are means ± s.e.m. (n = 10/group). Data analysed by repeated measures ANOVA.

For daily food intake, repeated measures ANOVA revealed highly significant effects of diet, time and their interaction (all *P* < 0.001; Figure 1a). There were no differences between CONT and CELL group daily intakes at any time point. GLUC, FOS and PECT groups all had lower daily intakes than the CONT group (the primary control group given 5% w/w insoluble fibre), with the differences being greatest during the first week but nonetheless present throughout the experiment (Figure 1a). Compared with the CELL group (the additional control group given 10% w/w insoluble fibre), daily intakes were significantly lower throughout (*P* < 0.001-0.05) in the GLUC group from day 2 and in FOS and PECT groups from day 1 (Figure 1a). Cumulative intake was significantly lower for rats fed GLUC (*P* < 0.05), FOS and PECT (both *P* < 0.001) than CONT and CELL, with no significant difference between CONT and CELL groups nor between GLUC, FOS and PECT groups (Table 2). Repeated measures ANOVA revealed significant effects on body weight of time (*P* < 0.001) and diet group (*P* < 0.01) with order of decreasing magnitude CELL > CONT > GLUC > FOS > PECT (Figure 1b). In view of the divergence in body weight between the groups, MRI values for total fat and lean mass are expressed as a percentage of the final body weight. Initial percentage total fat and lean were not different between the groups (Table 2). Compared with CONT, overall body weight gain was lower in GLUC (*P* < 0.01), FOS (*P* < 0.01) and PECT (*P* < 0.001) groups, body fat mass gain was lower in GLUC, FOS and PECT (all *P* < 0.001) but there was no difference in lean tissue mass gain (Table 2); differences in final body weight did not reach significance, final percentage total body fat was lower in GLUC (*P* < 0.05), FOS (*P* < 0.01) and PECT (*P* < 0.001) groups, and final percentage total lean tissue was higher in FOS (*P* < 0.05) and PECT (*P* < 0.01) groups (Table 2). Percentage total lean tissue was also greater in PECT than GLUC groups (*P* < 0.05), but there were no differences in fat or lean (changes in absolute mass or final percentage values) between CONT and CELL groups.

**Table 2:** **Cumulative food intake and changes in body weight and body composition**^**1 **^**in rats given different dietary fibres for 4 weeks**

	**Diet group**^**2**^				
	**CONT**	**CELL**	**FOS**	**GLUC**	**PECT**
Cumulative food intake (g)	543.5^a^ ± 22.8	566.2^a^ ± 16.1	448.6^b^ ± 15.1	488.5^b^ ±16.5	442.5^b^ ± 10.5
Body weight gain (g)	93.6^a^ ± 8.23	87.0^a^ ± 8.43	59.0^b^ ± 5.39	59.3^b^ ± 6.27	51.5^b^ ± 6.77
Body fat gain (g)	20.6^a^ ± 3.41	16.7^a^ ± 3.81	-0.15^b^ ± 2.07	2.25^b^ ± 1.78	-9.31^b^ ± 2.94
Lean tissue gain (g)	59.0 ± 5.22	59.4 ± 4.17	49.8 ± 4.34	41.3 ± 8.06	55.8 ± 4.91
Initial total body fat (%)	8.22 ± 0.63	7.60 ± 0.76	7.81 ± 0.57	8.21 ± 0.59	8.71 ± 0.87
Initial total body lean (%)	80.1 ± 0.63	80.5 ± 0.72	80.1 ± 0.65	80.1 ± 0.60	79.2 ± 0.87
Final total body fat (%)	10.4^a^ ± 0.86	9.26^a^ ± 1.03	6.81^b^ ± 0.73	7.71^b^ ± 0.58	6.08^b^ ± 0.46
Final total body lean (%)	77.5^a^ ± 0.93	78.7^a^ ± 0.96	80.7^b^ ± 0.81	79.1^a,b^ ± 1.35	82.1^b^ ± 0.43

^1^Total body fat mass and lean tissue mass measured by MRI.

^2^Diets were control (CONT, 5% w/w cellulose) or contained 10% w/w fibre as cellulose (CELL), fructo-oligosaccharide (FOS), oat beta-glucan (GLUC) or apple pectin (PECT); n = 10/group. Values are mean ± s.e.m. Within rows, values without a common letter are significantly different (*P* < 0.05).

**Figure 2:**
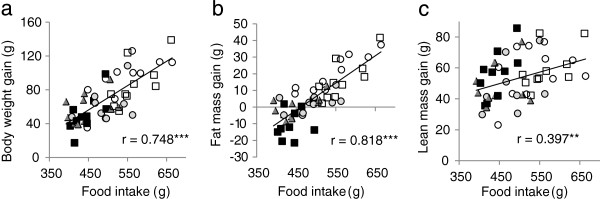
**Correlations between food intake and body weight and composition.** Relationships with cumulative food intake for **(a)** body weight gain, **(b)** total fat mass gain and **(c)** total lean mass gain for rats given diets containing different dietary fibres for 4 weeks. Diets contained 5% w/w cellulose (CONT, open circles) or 10% w/w fibre as cellulose (CELL, very light grey squares), oat beta-glucan (GLUC, light grey circles), fructo-oligosaccharide (FOS, dark grey triangles) or apple pectin (PECT, solid squares). **P < 0.01, ***P < 0.001; *r,* Pearson product–moment correlation coefficient.

Across all groups (n = 50) there were highly significant correlations between cumulative food intake and body weight gain (*P* < 0.001) and fat mass gain (*P* < 0.001) and a weaker correlation with lean tissue gain (*P* < 0.01) (Figures 2a-c).

### Plasma satiety hormone concentrations

**Figure 3:**
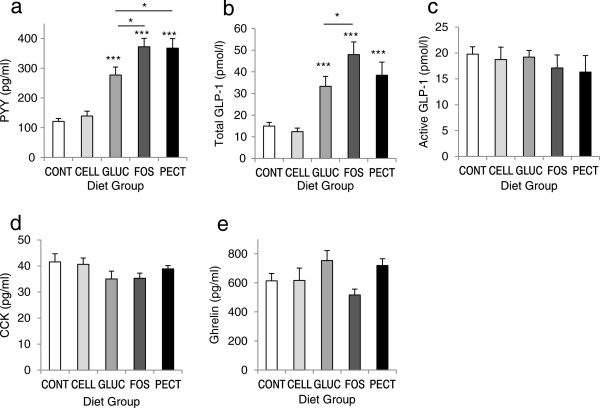
**Plasma hormone concentrations.** Plasma concentrations of **(a)** PYY, **(b)** total GLP-1, **(c)** active GLP-1, **(d)** CCK and **(e)** ghrelin in rats given diets containing different dietary fibres for 4 weeks. Diets contained 5% w/w cellulose (CONT) or 10% w/w fibre as cellulose (CELL), oat beta-glucan (GLUC), fructo-oligosaccharide (FOS) or apple pectin (PECT). Values are means ± s.e.m. (n = 10/group). Significant differences were detected by one-way ANOVA. *P < 0.05, ***P < 0.001 *vs* CONT, unless indicated.

Plasma concentrations of PYY and total GLP-1 were elevated in GLUC, FOS and PECT groups compared with CONT (all *P* < 0.001), and GLUC rats had lower PYY than FOS and PECT groups (both *P* < 0.05) and lower total GLP-1 than FOS rats (*P* < 0.05) (Figure 3a,b); there were no differences between CELL and CONT groups. There were no differences between any of the groups for active GLP-1, CCK or ghrelin concentrations (Figure 3c-e). Across all groups (n = 50) there were highly significant correlations between cumulative food intake and plasma concentrations of total GLP-1 (*r* = 0.516, *P* < 0.001) and PYY (*r* = 0.623, *P* < 0.001).

### Concentrations of fermentation products in caecum and colon contents

Total concentrations of fermentation products were significantly greater in the caecum of FOS, GLUC and PECT groups than CONT or CELL groups, greater in the colon of FOS and GLUC than CONT and CELL groups, and greater in the colon of PECT than CELL group (*P* < 0.05-0.001; Table 3). The main fermentation products detected were acetate, propionate, butyrate and succinate; lactate concentrations were low and were not significantly different between groups in caecum or colon, and concentrations of formate, iso-butyrate, valerate and iso-valerate were minimal (not reported). In caecal contents, compared with CONT, the FOS group had higher succinate (*P* < 0.001), lower acetate (*P* < 0.001) and lower propionate (*P* < 0.01), GLUC had higher succinate (*P* < 0.001) and lower acetate (*P* < 0.001), PECT had higher succinate (*P* < 0.01) and the CELL group was not different (Table 3). Butyrate concentrations did not differ from CONT, but were lower in PECT than FOS group (*P* < 0.01). Succinate was higher (*P* < 0.01) and acetate was lower (*P* < 0.001) in FOS and GLUC groups than in the PECT group. Similar group differences were seen in colonic contents where, compared with CONT, the FOS group had higher succinate (*P* < 0.001) and lower acetate (*P* < 0.001), GLUC had higher succinate (*P* < 0.001) and lower acetate (*P* < 0.001), PECT had higher succinate (*P* < 0.001) and the CELL group was not different (Table 3). There were no differences in colonic butyrate concentrations, but succinate was higher (*P* < 0.001) and acetate was lower (*P* < 0.01) in FOS and GLUC groups than in the PECT group.

**Table 3:** Concentrations (mmol/l) of fermentation products in caecum and colon contents of rats given different dietary treatments

	**Diet group **^**1**^				
	**CONT**	**CELL**	**FOS**	**GLUC**	**PECT**
**Caecum**					
*Concentrations (mmol/l)*					
Total	68.9 ± 7.01^a^	61.2 ± 7.79^a^	115.9 ± 10.80^b,c^	131.7 ± 6.82^c^	97.7 ± 6.88^b^
Acetate	47.6 ± 2.72^a^	41.2 ± 3.14^a^	21.7 ± 3.51^b^	23.8 ± 3.38^b^	49.2 ± 6.00^a^
Propionate	8.6 ± 0.58^a,b^	6.7 ± 0.48^b,c^	4.0 ± 0.89^c^	11.9 ± 1.88^a^	7.8 ± 0.70^b,c^
Butyrate	7.1 ± 0.58^a,b^	8.0 ± 1.37^a,b^	10.3 ± 2.29^a^	7.5 ± 1.48^a,b^	3.9 ± 0.84^b^
Succinate	5.3 ± 4.70^a^	6.2 ± 3.52^a^	74.2 ± 11.03^b^	88.5 ± 8.93^b^	36.8 ± 7.14^c^
Lactate	1.3 ± 0.92	0.0	5.7 ± 4.64	0.0	0.0
*Proportional concentrations*					
Acetate	0.69	0.67	0.19	0.18	0.50
Propionate	0.12	0.11	0.03	0.09	0.08
Butyrate	0.10	0.13	0.09	0.06	0.04
Succinate	0.08	0.10	0.64	0.67	0.38
Lactate	0.02	0.00	0.05	0.00	0.00
**Colon**					
*Concentrations (mmol/l)*					
Total	63.5 ± 6.53^a,b^	52.0 ± 6.51^b^	121.3 ± 4.51^c^	131.0 ± 5.41^c^	79.7 ± 8.63^a^
Acetate	40.9 ± 4.59^a^	33.4 ± 2.96^a^	15.7 ± 1.85^b^	16.4 ± 3.46^b^	32.4 ± 3.97^a^
Propionate	7.7 ± 0.77	6.4 ± 0.74	3.6 ± 0.99	5.0 ± 1.50	6.6 ± 1.20
Butyrate	5.6 ± 0.82	8.2 ± 3.08	5.4 ± 1.30	5.3 ± 1.74	2.9 ± 0.70
Succinate	6.7 ± 3.41^a^	3.0 ± 0.80^a^	92.0 ± 9.35^b^	104.3 ± 6.73^b^	37.8 ± 8.70^c^
Lactate	2.6 ± 1.98	1.0 ± 0.79	4.6 ± 3.87	0.0	0.0
*Proportional concentrations*					
Acetate	0.64	0.64	0.13	0.13	0.41
Propionate	0.12	0.12	0.03	0.04	0.08
Butyrate	0.09	0.16	0.04	0.04	0.04
Succinate	0.11	0.06	0.76	0.80	0.47
Lactate	0.04	0.02	0.04	0.00	0.00

^1^Diets were control (CONT) or contained 10% w/w fibre as cellulose (CELL), fructo-oligosaccharide (FOS), oat beta-glucan (GLUC) or apple pectin (PECT). Values are means ± s.e.m. (n = 10/group). Within rows, values without a common letter are significantly different (*P* < 0.05).

### Gene expression for SCFA and succinate receptors and for satiety hormones in ileum and colon tissue

Relative to CONT group, ffar2 gene expression was increased in the distal ileum of the FOS group (*P* < 0.01; Table 4); ffar3 gene expression was decreased in proximal colon of FOS and PECT groups (*P* < 0.05-0.01); sucnr1 gene expression was increased in distal ileum and proximal colon of CELL group (*P* < 0.05-0.01); GLP-1 gene expression was decreased in distal ileum of PECT group (*P* < 0.05); PYY gene expression was decreased in distal ileum of PECT group and in proximal colon of CELL group (*P* < 0.05); and relative expression of all other gene/tissue combinations was not significantly different (Table 4).

**Table 4:** Gene expression in distal ileum and proximal colon for experimental diet groups relative to control (CONT) group

**Gene**^**1**^	**Group**^**2**^	**Gene expression**^**3**^	
		**Distal ileum**	**Proximal colon**
ffar2	CELL	1.16 (0.12-5.16)	1.24 (0.20-3.14)
	FOS	2.15 (0.73-7.05)**	1.02 (0.33-3.10)
	GLUC	1.07 (0.33-3.81)	1.07 (0.43-2.97)
	PECT	1.23 (0.42-7.31)	0.63 (0.13-2.48)
ffar3	CELL	1.40 (0.15-14.72)	0.68 (0.14-3.58)
	FOS	1.11 (0.21-9.44)	0.42 (0.06-2.39)*
	GLUC	0.49 (0.02-4.91)	0.63 (0.10-7.68)
	PECT	1.15 (0.13-11.39)	0.42 (0.09-1.85)**
sucnr1	CELL	3.59 (0.17-33.62)*	4.13 (0.47-52.44)**
	FOS	0.70 (0.03-11.53)	0.82 (0.06-8.85)
	GLUC	1.75 (0.06-23.59)	1.65 (0.11-56.72)
	PECT	0.95 (0.01-16.54)	0.79 (0.04-14.45)
GLP-1	CELL	0.81 (0.34-2.01)	0.71 (0.13-4.48)
	FOS	0.79 (0.23-1.93)	1.54 (0.35-9.73)
	GLUC	0.78 (0.35-1.69)	1.82 (0.43-10.01)
	PECT	0.53 (0.03-1.71)*	1.18 (0.15-8.77)
PYY	CELL	0.97 (0.46-2.50)	0.62 (0.30-1.32)*
	FOS	0.91 (0.24-2.74)	0.78 (0.32-3.71)
	GLUC	0.79 (0.32-2.16)	1.11 (0.30-3.18)
	PECT	0.40 (0.00-1.75)*	0.71 (0.11-2.70)

^1^*Abbreviations* are: *ffar* free fatty acid receptor, *sucnr* succinate receptor, *GLP-1* glucagon-like peptide-1, *PYY* peptide tyrosine tyrosine.

^2^Groups were given diets containing 10% w/w fibre as cellulose (CELL), fructo-oligosaccharide (FOS), oat beta-glucan (GLUC) or apple pectin (PECT); n = 10/group.

^3^Values are medians (with 95% C.I.) as determined by REST-2009 [32]. *P < 0.05, **P < 0.01.

## Discussion

This experiment has shown how three very different soluble fermentable dietary fibres, beta-glucan, FOS and pectin, all similarly decreased voluntary food intake, weight gain and adiposity in normal healthy young adult male rats over a 4-week period, and the associated increased plasma concentrations of PYY and GLP-1 were strongly indicative of increased satiety. While these data demonstrate an animal model of fibre-induced satiety, the dietary inclusion rate (10% w/w, or 6.6 mg/kJ) was approximately twice the US recommended daily fibre intake of 3.3 mg/kJ for men [33,34] and further work is required to translate the findings to humans.

Whereas 10% w/w insoluble fibre cellulose had no effect on food intake compared with the control group, the same inclusion rate of the three soluble dietary fibres each had similar effects, decreasing cumulative intake by 10-19% over 4 weeks and daily intakes by ~12% after an initial week of adjustment. Although the immediate decreases in intake following introduction of the high fibre diets may have reflected some initial unfamiliarity and low palatability to the rats, the consistently lower daily intakes thereafter once they had become accustomed to the diets were more likely indicative of increased satiety since they were associated with increased circulating concentrations of two important satiety hormones. The three soluble dietary fibres specifically increased concentrations of total GLP-1 and PYY in plasma samples taken in the early part of the light phase at the end of the trial, indicative of raised tonic secretion of these hormones, and the magnitude of the increases were similar to those associated with increased satiety in other rat models [16,35]. Predictably, changes in active GLP-1 were not detected since it only has a 1-2 minute half-life and samples were not taken immediately after feeding episodes; nonetheless, measurement of total GLP-1 includes both the intact hormone and its primary metabolite and thereby provides an accurate indication of overall GLP-1 secretion [36]. Tonic plasma concentrations of ghrelin and CCK were unchanged. This was perhaps not surprising since ghrelin is secreted in the stomach and CCK is produced by I-cells that are located mainly in the duodenum and jejunum whereas the presence of indigestible dietary fibre in the gut is most likely to be detected nearer to where it is fermented in the large intestine. In line with this argument, PYY and GLP-1 are both secreted from L-cells situated mainly in the distal small intestine and proximal large intestine [18]. Our data are consistent with findings in rats fed dietary resistant starch where total GLP-1 and PYY were up-regulated in a sustained day-long manner through fermentation [35]. It is inferred that significant satiety-inducing effects of dietary fibre only manifest themselves after several days of consistently increased fibre intake and it is tempting to speculate that this reflects a minimum exposure time for the underlying chronic changes in the gut environment and in tonic gut satiety hormone secretion to develop.

The reductions in food intake on the three diets containing soluble fermentable fibre were associated with significant reductions in body weight gain and it is noteworthy that this was attributable to the arrested accumulation of fat with lean tissue growth maintained. Both body weight gain and fat mass gain were closely correlated with the cumulative food intake (Figure 2). These findings add important total body composition data to existing reports of dietary oat beta-glucan decreasing epididymal fat pad weights in growing rats [10] and of dietary FOS decreasing fat pad mass in rats [10,13] and lend support to the notion that fermentable dietary fibre has potential as a tool for body weight and body fat regulation through its effects on satiety. While differences in eating behaviour, physical activity and heat expenditure (from increased fermentation) may also have contributed to the observed differences between the soluble fibre-fed rats and controls, the overall outcome was a similar decrease in food intake, body weight and body fat in all three soluble fibre-fed groups.

Soluble dietary fibres may influence satiety by virtue of their physicochemical properties, for example those with high viscosity such as pectin and beta-glucan tend to slow the rate of gastric emptying and overall gut transit time, which can lead to reduced intakes [10,14,15]. However the present intake responses were also shown with FOS, which has negligible viscosity [10]. The common feature of the three fibres studied herein was their fermentability in the large intestine, resulting in greatly increased total concentrations of fermentation products in the caecum and colon contents. The highest concentration was that of succinate, whereas dietary studies in the literature appear rarely to report succinate concentrations, concentrating rather on the main three SCFAs. Succinate is produced by intestinal bacterial species of the *Bacteroides* and *Clostridium* genera [21], numbers of *Bacteroides* have been shown to increase in rats given dietary inulin and FOS [37] and increased caecal succinate concentrations of comparable magnitude to those reported herein have previously been reported in rats given 6% FOS [38] or 5% pectin [39]. It appears from these latter studies and from the present one that the production of large amounts of succinate is favoured when rats are given a rapidly soluble and fermentable fibre source in a purified diet and when the protein source is a highly digestible one such as casein; this is thought to be because a lack of nitrogen relative to the carbohydrate availability in the large intestine promotes high succinate concentrations [38]. Moreover, succinate is absorbed by the intestinal mucosa more slowly than other SCFAs [40], which would lead to its relative accumulation. However, it is unlikely that the increased succinate *per se* elicited the satiety response in our model since succinate concentrations were lower in PECT than GLUC or FOS groups, yet the satiety response was similar. Furthermore, although increased luminal succinate was associated with increased PYY and GLP-1 secretion in the fermentable fibre-fed groups, there was no evidence for increased succinate signalling to the gut enteroendocrine cells via its receptor sucnr1. Indeed, the only increase in sucnr1 gene expression was unexpectedly seen in the CELL group, in the absence of elevated luminal succinate and without changes in PYY and GLP-1 secretion. It is unknown whether succinate activates the SCFA receptors, ffar2 and ffar3 [41].

After succinate, in terms of relative concentrations, acetate was the dominant fermentation product in all three fermentable fibre groups, but whereas concentrations of propionate were greater than butyrate in PECT and GLUC groups, the reverse was observed in the FOS group. The differences in fermentation products between the soluble fibres may reflect different patterns of fermentation and/or different rates of fermentation and turnover, which the present measurements at a single time point would not have detected. Nonetheless, given the similar levels of satiety observed in all groups, it is hard to attribute relevance of these SCFA differences to signalling satiety.

It was perhaps surprising that there were no increases in concentrations of the main three SCFAs in caecum and colon contents of rats fed the fermentable fibres compared with controls, and indeed concentrations were unexpectedly lower for acetate in FOS and GLUC groups and for propionate in the FOS group. There may be a number of reasons for this, apart from the overwhelming dominance of the succinate: for example, measuring concentrations at a single time point would have given no indication of biologically significant changes in rates of turnover. Nonetheless, the volume of contents and hence the total pool of SCFAs would have been increased in the fermentable fibre groups because the large intestine was visibly enlarged. This observation was supported by measurements of increased caecum and colon weights in rats given 10% PECT in a separate experiment (Adam *et al.*, unpublished results) and is in agreement with published data on rats fed the dietary fibre inulin [42]. Although the caecum is the primary site for dietary fibre fermentation, SCFA concentrations and group differences were similar in colon and caecum contents. There was also visible leakage of caecal contents back into the distal ileum, indicating that enteroendocrine L-cells in both the distal ileum and proximal colon would have been exposed to SCFAs, from where satiety responses may have been elicited, and a recent report indicates that L-cell numbers are fairly evenly distributed along the rat jejunum, ileum and colon [43].

Whereas a recent review concludes overall that SCFAs may not play a role in appetite regulation [44], *in vitro* evidence shows that they can trigger GLP-1 release from cultured colonic L-cells via the SCFA receptors ffar2 and ffar3 [25] and stimulate GLP-1 (proglucagon) mRNA expression in cultured enteroendocrine STC-1 cells [35]. Some *in vivo* evidence indicates that oral administration of butyrate or propionate decreases food intake and butyrate stimulates GLP-1 and PYY secretion in mice, but these were ffar3-deficient animals indicating that ffar3 is not involved in SCFA stimulation of these gut satiety hormones [45]. The present data are consistent with this since we found no evidence for increased ffar3 gene expression and indeed there was even a decrease in expression in the proximal colon of FOS and PECT groups (Table 4). Similarly we found little evidence for increased gene expression for ffar2, the one exception being an increase in the distal ileum of FOS-fed rats. The absence of increased gene expression found here for PYY and GLP-1 in distal ileum and proximal colon, despite the greatly increased plasma concentrations of these hormones, was another apparently anomalous finding and gene expression for both was even decreased in the PECT group. Since PYY and GLP-1 are secreted from intestinal L-cells, the increased plasma concentrations must reflect either increased secretory activity per cell or increased number of L-cells. The apparent anomalies are most likely attributable to an overall increase in L-cell number along the enlarged intestines, which was not detectable here since the real time PCR method measures expression per mg tissue. The same PECT diet given for 4 weeks to adult male rats in a separate experiment increased the weights and lengths of both colon and small intestine (Adam *et al.*, unpublished results). This is not without precedent since a doubling of the L-cell population in the rat intestinal mucosa occurs with no change in L-cell density due to general gut hypertrophy both after Roux-en-Y gastric bypass and in the Zucker Diabetic Fatty rat model [43,46]. These authors also report increased plasma GLP-1 concentrations in their models, associated with the increased L-cell number and overall increased proglucagon (GLP-1) gene expression (by *in situ* hybridisation) without any increase in gene expression per cell. Other reports of increased L-cell differentiation and number in the proximal colon of rats fed fermentable indigestible carbohydrate lend support to this argument [13,23]. Altogether the present data are consistent with increased circulating PYY and GLP-1 concentrations arising from overall increased numbers of L cells along the ileum and colon exposed to increased amounts of fermentation products, but further mechanistic investigations need to acknowledge the potential influence of gut hypertrophy in this model.

## Conclusions

These data show that sustained daily intake of diverse types of soluble dietary fibre increases satiety and decreases overall food intake, weight gain and adiposity, associated with increased circulating GLP-1 and PYY and increased hindgut fermentation. Moreover it appears that the presence of soluble fibre is more important than the source of soluble fibre for reducing diet intake and body weight gain. Therefore, the study demonstrates an animal model of dietary fibre-induced satiety that may be used for further investigation of underlying mechanisms and provides proof of principle worthy of investigation in humans towards a natural means of body weight regulation.

## Abbreviations

FOS: Fructo-oligosaccharide; CCK: Cholecystokinin; PYY: Peptide tyrosine tyrosine; GLP-1: Glucagon-like peptide-1; SCFA: Short chain fatty acid; Ffar: Free fatty acid receptor; Sucnr: Succinate receptor; REST: Relative expression software tool.

## Competing interests

The authors declare that they have no competing interest.

## Authors’ contributions

CLA and AWR formulated the research question. CLA took primary responsibility for designing the study, analysing the data and writing the article. Sample collection was carried out by CLA, PAW, LMT and AWR; radioimmunoassays and method description by PAW; SCFA analyses, description and interpretation by MJD, AJR and SWG; qPCR analyses, description and interpretation by MJD, LMT, KG and AWR; AWR and SWG contributed to the intellectual content of the manuscript and all co-authors approved the final version.
